# Reliability Assessment of MEMS Gyroscopes via Dual-Mechanism Synergistic Degradation: A Generalized Linear Model with Physics-Informed Wiener Processes

**DOI:** 10.3390/s26123774

**Published:** 2026-06-12

**Authors:** Pengbin Yang, Zhen Liu, Yuhang Liang, Xinfeng Guo, Hang Geng

**Affiliations:** School of Automation Engineering, University of Electronic Science and Technology of China, Chengdu 611731, China; yangpb_eric@std.uestc.edu.cn (P.Y.); 202311060915@std.uestc.edu.cn (Y.L.); 202411060928@std.uestc.edu.cn (X.G.); Hang.Geng@uestc.edu.cn (H.G.)

**Keywords:** MEMS, reliability assessment, multi-mechanism

## Abstract

As the core sensor of inertial measurement units, the reliability of Micro-Electro-Mechanical Systems (MEMS) gyroscopes is critical for long-term navigation and motion control applications. To bridge the mechanism-data gap in MEMS multi-mechanism degradation modeling, this paper proposes a physics-informed dual-indicator reliability assessment framework based on Wiener processes. Two degradation indicators under consideration are frequency-related degradation caused by stiffness degradation and Q-factor degradation caused by damping degradation, for which corresponding physics-embedded stochastic degradation models are formulated. The two indicators are normalized and fused through a generalized weighted limit state function, where failure is defined as gyroscope-level performance failure. Closed-form reliability expressions are derived for linear limit states, while Monte Carlo simulation is used for nonlinear cases. Reduced-order multiphysics simulation cases, including a double-ended fixed beam and a cantilevered MEMS mass block, are used to demonstrate the mechanism-to-indicator-to-reliability modeling procedure. The results show that the proposed dual-indicator framework provides more balanced reliability assessment than single-indicator analysis under the simulation setting. The proposed method offers an alternative mechanism-informed approach for reliability analysis and lifetime prediction of other MEMS devices.

## 1. Introduction

As the core component of inertial measurement units (IMUs), Micro-Electro-Mechanical Systems (MEMS) gyroscopes are widely employed in critical domains such as aerospace, autonomous driving, and even deep-sea drilling, serving as essential devices for achieving attitude estimation, motion control, and precision navigation [1,2,3,4,5]. As application scenarios continue to expand, MEMS gyroscopes are confronted with increasingly complex operating environments, where factors such as shock, vibration, and thermal cycling may induce device failures. Even minor malfunctions can lead to system-level paralysis, causing significant safety accidents and economic losses. Therefore, conducting failure analysis and reliability assessment research on MEMS gyroscopes and establishing accurate reliability characterization models hold important engineering significance and theoretical value for ensuring the stable operation of critical equipment [6,7].

Research on MEMS reliability analysis and lifetime prediction can be approached from two perspectives: (1) quantitative reliability modeling methods based on lifetime data or performance degradation trajectories; and (2) qualitative analysis methods based on environmental reliability testing.

Quantitative modeling methods aim to develop general approaches for remaining useful life and reliability prediction, primarily dividing into two branches of stochastic process modeling and deep learning modeling, with some studies attempting to integrate the advantages of both approaches. In terms of stochastic process modeling, Bu et al. proposed a multivariate adaptive Brownian motion-generalized particle filter framework, introducing polynomial drift terms to accommodate complex nonlinear degradation patterns, thereby improving the average prediction accuracy of remaining useful life [8]. To address the narrow applicability of conventional stochastic process models, Wu et al. proposed a mechanism-equivalence-based Tweedie exponential dispersion process [9]. Wu et al. developed a two-parameter Wiener degradation process by identifying dominant failure modes, predicting the remaining useful life of wet friction components in traditional systems [10]. Dong et al. developed an adaptive Wiener process degradation model that accounts for the effects of imperfect maintenance on reliability analysis and lifetime prediction [11]. In recent years, remarkable achievements have been made in deep learning modeling [12], Wang et al. developed a deep residual shrinkage network–temporal convolutional network (DRSN-TCN) prediction framework, enhancing the performance of bearing reliability assessment [13]. Li et al. employed a convolutional neural network (CNN) model for feature extraction, utilized a bidirectional long short-term memory (Bi-LSTM) model to capture the time series degradation characteristics of engineering features, and performed remaining useful life prediction through regression [14]. Jiang et al. proposed a hybrid remaining useful life prediction model based on extended long short-term memory (xLSTM) and transformer to address the challenge that traditional deep learning models struggle to simultaneously capture local temporal features and global degradation trends when processing degradation health indicators [15]. In recent years, hybrid modeling methods combining stochastic processes and neural networks have gradually emerged. Chen et al. proposed using a trained long short-term memory (LSTM) neural network as the degradation trend function for Wiener process degradation models, and derived closed-form expressions for remaining useful life [16].

Qualitative analysis methods, with environmental reliability testing as the core means, focus on identifying failure modes and analyzing failure mechanisms. For example, Li et al. investigated the packaging and functional failure mechanisms that may occur in three-axis gyroscopes under shock [17]. Xu et al. conducted an in-depth study on the influence mechanism of gas leakage on the quality factor (Q-factor) for wafer-level MEMS packaging, and derived a quantitative relationship between the Q-factor and the number of leaked gas molecules [18]. Wang et al. studied the structural response and failure mechanism of MEMS vibrating ring gyroscopes (VRG) under high-g shock, and established physical models for fracture-sensitive positions and delamination failure under different shock conditions [19]. Liu et al. pointed out in their research that the relaxation of residual stress in butterfly resonant gyroscopes generates axial forces, leading to resonant frequency drift and, ultimately, a decline in sensitivity [20]. Cheng et al. correlated fatigue life with parameters such as average crack length, maximum stress, resonant frequency, and stiffness through multi-scale analysis, revealing the inherent law that fatigue of silicon microfilms causes stiffness degradation and thus resonant frequency drift [21,22,23]. Sadurska et al. introduced an auxiliary simulation method to investigate the relationship between MEMS operating performance and temperature, pointing out that its mean time to failure exhibits an exponential relationship with temperature and is inversely proportional to current density [24]. Satheesh et al. generated the probability distribution of the failure load of the tested structure using the weakest link parameters of MEMS polysilicon micro-cantilevers, clarifying that the cavity, at the position of the maximum bending stress, has no significant effect on the failure load [25]. Such studies provide important support for understanding the physical nature of MEMS gyroscope failure, but they have realized quantitative prediction of remaining life and reliability level, making it difficult to meet the demand for accurate assessment in engineering practice.

In addition to reliability modeling and failure mechanism analysis, recent reviews have summarized MEMS gyroscopes from the perspectives of operation modes, structural forms, processing technologies, and performance indices, showing that gyroscope performance is determined not only by resonator geometry but also by coupled mechanical, electrical, and control characteristics [3,26]. For symmetric MEMS gyroscopes, ideal mode matching between the drive and sense modes is generally required to achieve high sensitivity and stable scale factor; however, processing limitations and structural imperfections often introduce frequency split and mode mismatch [27]. Therefore, the relative drift between the drive and sense resonant frequencies is often more directly related to gyroscope-level performance than the absolute drift of a single resonant frequency [28]. Meanwhile, Q-factor and Q-factor mismatch are also critical because they are associated with energy dissipation, damping coupling, zero-rate output, and bias instability [29]. Temperature variation further affects resonant frequency, frequency split, demodulation phase error, scale factor, and bias stability [30,31]. These studies indicate that frequency-related degradation and Q-factor-related degradation are physically meaningful indicators for MEMS gyroscope reliability assessment.

It should be noted that the failure of MEMS is not the result of a single mechanism but the combined action and mutual influence of multiple physical, chemical, and mechanical mechanisms [32,33]. During the service of MEMS, there often exist multiple degradation processes, such as friction and wear (common in micro-engine [34]), fatigue cracking [35,36], and packaging aging [17]. Traditional reliability analysis evaluates overall system reliability through series or parallel models. However, such assumptions neglect the coupling effects between different mechanisms [37]. Therefore, research on multi-failure mechanism coupling problems represents a frontier and hotspot in the MEMS reliability field. Zhang et al. proposed a comprehensive method utilizing multiple dependent competing failure processes (DCFP) models, establishing an overall reliability assessment model that simultaneously considers structural failure and performance degradation [38]. Liu et al. proposed a multi-sensor fusion system that utilizes an artificial bee colony algorithm-optimized backpropagation network to achieve online prediction of machine tool remaining useful life [39]. Physics-based degradation models have also attracted attention. Yin et al. proposed a PLT-GRU method that captures physical priors in the degradation process, improving the accuracy and interpretability of few-shot lithium-ion battery lifetime prediction [40]. Wu et al. combined physics-driven digital twins with ensemble learning to enhance the accuracy and adaptability of remaining useful life prediction [41].

Overall, early multivariate degradation process models mostly employed stochastic processes and regression models to describe the degradation of correlated indicators but lacked clear physical explanation; conversely, pure physical analysis struggles to explain the random fluctuations present in measured degradation data. Therefore, existing research still cannot address the “mechanism-data gap” problem in MEMS multivariate synergistic degradation.

To address the aforementioned issues, the core contributions of this paper are as follows: (1) A physics-informed dual-indicator reliability assessment framework is proposed for MEMS gyroscopes. Unlike single-indicator reliability analysis, the proposed framework integrates frequency-related degradation and Q-factor degradation into a unified generalized limit state function, thereby enabling system-level performance reliability assessment under two mechanism-specific degradation channels. (2) Two differentiated stochastic degradation models are constructed by embedding physical degradation characteristics into Wiener processes. A Wiener process with linear drift is used to describe frequency-related degradation, while a physics-informed Wiener process with a nonlinear physical degradation trajectory is used to describe Q-factor degradation. (3) Both analytical and simulation-based reliability solutions are provided. A closed-form reliability expression is derived for the linear limit state, while a Monte Carlo procedure is developed for nonlinear limit states. Reduced-order multiphysics simulation cases are used to demonstrate the mechanism-to-indicator-to-reliability modeling procedure.

The remainder of this paper is organized as follows: Section 2 elaborates on the degradation causes of two key macroscopic parameters—resonant frequency and quality factor (Q-factor)—and, based on this, analyzes the core problems to be addressed in this paper; Section 3 systematically proposes a reliability modeling method for synergistic degradation of resonant frequency and Q-factor based on generalized linear models; Section 4 presents parameter estimation methods based on maximum likelihood estimation and numerical optimization for the established physics-informed performance degradation models; Section 5 demonstrates the feasibility of the proposed method through reduced-order simulation cases; Section 6 discusses the applicability and limitations of the proposed framework; and Section 7 summarizes the main conclusions and future research directions.

## 2. Problem Analysis

The failure mechanism of MEMS gyroscopes fundamentally involves irreversible degradation of their dynamic performance. Resonant frequency and quality factor (hereafter referred to as Q-factor) are two core macroscopic physical quantities characterizing this dynamic performance. As noted in the introduction, existing research often isolates the degradation of single metrics or focuses solely on statistical patterns, resulting in a disconnect between mechanisms and data. To achieve mechanism-guided reliability analysis, the primary step is to trace the degradation of macro-performance metrics back to fundamental changes in physical parameters. This section aims to accomplish this task, laying the physical foundation for subsequent development of a mechanism-embedded synergistic degradation model.

### 2.1. Degradation Analysis of Two Key Performance Parameters

**Figure 1:**
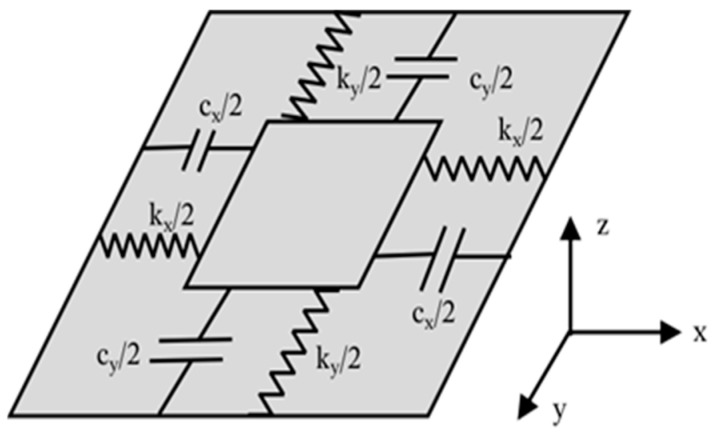
Schematic diagram of the coupled drive–sense dynamics of a MEMS gyroscope.

MEMS gyroscopes are quintessential inertial devices that leverage the fundamental Coriolis effect to achieve high-precision angular velocity sensing in demanding environments. Capacitive MEMS gyroscopes utilize comb-shaped capacitors as sensing elements, with their movable structure comprising core components, such as mass blocks, drive teeth, and sensing teeth. Under stable voltage drive, the mass block maintains resonant motion in the drive direction. When angular velocity input occurs along the Z-axis, Coriolis forces are generated in the detection direction, with their magnitude indicating the angular velocity. This motion principle can be abstracted as a second-order “spring-damper-mass block” system, as illustrated in Figure 1. MEMS gyroscopes generally involve coupled drive and sense modes, and the dynamics of the movable structure can be expressed in a generalized two-degrees-of-freedom form as:

$$M\ddot{u}(t)+C\dot{u}(t)+Ku(t)=F(t),u(t)=[x\left(t\right),y\left(t\right)]$$

where $u(t)=[x\left(t\right),y\left(t\right)]$ denotes the displacement vector, x(t) and y(t) denote the driving and sense mode displacements, respectively; M, C, and K represent the mass, damping, and stiffness matrices, respectively; and F(t) represents the source term. In practical devices, structural imperfections, process variations, residual stress, and environmental perturbations may change stiffness and damping terms, resulting in frequency mismatch, quadrature error, cross-axis coupling, and mode-matching drift.

To determine the contribution of stiffness and damping degradation to the degradation of gyroscope performance, this study first considers the drive direction as a reduced-order representative model. The corresponding dynamic equation is written as:

(1) $$\begin{array}{c} m_{x}\ddot{x}\left(t\right)+c_{x}\dot{x}\left(t\right)+k_{x}x\left(t\right)=f_{0}sin\left(\omega t\right) \end{array}$$

where $m_{x}$ is the equivalent mass in the drive direction; $c_{x}$ is the equivalent damping coefficient in the drive direction; $k_{x}$ is the equivalent stiffness in the drive direction; and $f_{0}sin\left(\omega t\right)$ represents the harmonic driving force.

#### 2.1.1. Resonant Frequency Degradation: Stiffness Degradation Dominates

Performing a Laplace transform on (1) and solving for the mechanical response amplitude in open-loop operation yields:

(2) $$\begin{array}{c} A_{x}=\frac{\frac{f_{0}}{m_{x}}}{\sqrt{\omega^{4}+\left(4\xi_{x}^{2}-2\right)\omega_{x}^{2}\omega^{2}+\omega_{x}^{4}}} \end{array}$$

where ω is the resonant frequency; $\omega_{x}$ is the natural frequency in the drive direction; and $\xi_{x}$ is the damping ratio in the drive direction, with $\xi_{x}=c_{x}/(2m_{x}\omega_{x})$. When the MEMS gyroscope operates in resonance, $A_{x}$ reaches its maximum, while $f\left(\omega \right)=\omega^{4}+\left(4\xi_{x}^{2}-2\right)\omega_{x}^{2}\omega^{2}+\omega_{x}^{4}$ reaches its minimum. Solving for the extrema of $f\left(\omega \right)$ yields the resonant frequency:

(3) $$\begin{array}{c} \omega =\omega_{x}\sqrt{1-2\xi_{x}^{2}} \end{array}$$

Substituting the expression for the damping ratio $\xi_{x}=c_{x}/(2m_{x}\omega_{x})$ and the expression for the natural frequency $\omega_{x}=\sqrt{k_{x}/m_{x}}$ into (3) allows the resonant frequency expression to be further written as:

(4) $$\begin{array}{c} \omega =\sqrt{1-{\left(\frac{c_{x}}{m_{x}\omega_{x}}\right)}^{2}}\sqrt{\frac{k_{x}}{m_{x}}} \end{array}$$

Assuming the equivalent mass $m_{x}$ remains constant during long-term storage or operation, we analyze the influence of stiffness $k_{x}$ and damping $c_{x}$ on the resonant frequency ω. Their relative sensitivity coefficients are:

(5) $$\begin{array}{c} \frac{\left|S_{c_{x}}\right|}{\left|S_{k_{x}}\right|}=\frac{\frac{c_{x}^{2}}{k_{x}m_{x}-c_{x}^{2}}}{\frac{k_{x}m_{x}}{2\left(k_{x}m_{x}-c_{x}^{2}\right)}}=2\cdot \frac{c_{x}^{2}}{k_{x}m_{x}}=8\xi^{2} \end{array}$$

Among these, $\xi =c_{x}/2\sqrt{k_{x}m_{x}}$ represents the damping ratio. When ξ is small, it indicates that ω is more sensitive to changes in $k_{x}$. Conversely, when ξ is large, it suggests that ω is more responsive to variations in $c_{x}$. In practical engineering applications, MEMS resonators typically operate in an underdamped state. Under such conditions, the damping ratio becomes extremely small, such that $\left|S_{c_{x}}\right|/\left|S_{k_{x}}\right|\ll 1$. Consequently, $k_{x}$ exerts a significantly greater influence on ω than $c_{x}$. Therefore, it can be concluded that the primary cause of the resonant frequency drift ω is the degradation of stiffness $k_{x}$.

#### 2.1.2. Q-Factor Degradation: Dominated by Increased Damping

Next, we analyze the fundamental mechanism of Q-factor degradation. As previously noted, the Q-factor is a key parameter characterizing the energy dissipation efficiency of a resonant system. It is defined as the ratio of the total stored energy to the energy dissipated per cycle, multiplied by 2π. For the system shown in (1), its Q-factor can be explicitly expressed as:

(6) $$\begin{array}{c} Q=2\pi \frac{\frac{1}{2k_{x}A_{x}^{2}}}{\pi c_{x}\omega A_{x}^{2}}=\frac{wm_{x}}{c_{x}} \end{array}$$

Equation (6) clearly demonstrates that the Q-factor is inversely proportional to the damping coefficient $c_{x}$. Any physical mechanism that increases $c_{x}$ will directly cause a decrease in Q-factor. These mechanisms include air damping, thermoelastic damping, and anchor losses, among others [42,43]. Overall, the damping in MEMS resonant systems can be simplified into a rarefied gas damping term related to gas pressure and other structural losses, namely:

(7) $$\begin{array}{c} c_{x}=c_{gas}+c_{struc} \end{array}$$

where $c_{gas}=c\left(P\right)$ represents gas-related damping, dependent on the pressure P within the sealed cavity, while $c_{struc}$ denotes other structural losses. Research by Schiwietz et al. indicates that when the pressure within the sealed cavity exceeds $10^{-2}\;mbar$, an increase in pressure significantly reduces the Q-factor [43]. This demonstrates that gas damping becomes the dominant factor under such conditions.

### 2.2. The Core Problem Addressed in This Paper

The above analysis identifies two degradation processes with distinct dominant physical origins: frequency-related degradation caused by stiffness degradation and Q-factor degradation caused by damping degradation. However, how do they synergistically interact within a MEMS device, ultimately leading to system-level failure? Traditional single-parameter threshold methods cannot capture this synergistic effect, often resulting in overly conservative or aggressive reliability predictions. Therefore, this study faces three key challenges:

1. Collaborative characterization: how can a unified mathematical framework be established to map two degradation processes with distinct physical origins and varying trends onto a comprehensive system limit state function, enabling collaborative reliability analysis?
2. Mechanism-linked modeling: how can the physical characteristics derived from the above analysis be effectively embedded into their respective stochastic degradation models, enabling consistent parameter estimation?
3. Quantitative analysis: how can the time-varying contributions of both mechanisms to overall failure be quantified to address potential nonlinear cumulative effects during degradation?

## 3. Reliability Analysis Method for MEMS Gyroscopes with Synergistic Degradation of Resonant Frequency and Q-Factor

This paper proposes applying generalized linear models to characterize synergistic relationships among different failure mechanisms in MEMS gyroscopes. By mapping the synergistic relationships of physical failure mechanisms onto a unified probabilistic space, this overcomes the potentially conservative or aggressive results that may arise when using single parameters for threshold determination and reliability analysis.

### 3.1. Limit State Function for Two-Mechanism Synergistic Effects

The two typical types of degradation in MEMS—resonant frequency and Q-factor degradation—arise from different causes but synergistically interact within MEMS resonant systems, collectively leading to performance degradation or even product failure in MEMS gyroscopes. To describe the synergistic degradation effect of these two mechanisms, we first define a limit state function that integrates both synergistic degradation mechanisms:

(8) $$\begin{array}{c} g\left(X,t\right)=\left|\lambda_{1}\right|{\tilde{f}}^{m}\left(t\right)+\left|\lambda_{2}\right|{\tilde{Q}}^{n}\left(t\right) \end{array}$$

where X is the vector containing all random variables (e.g., drift coefficients and diffusion coefficients in two Wiener processes); $\tilde{f}\left(t\right)$ is the covariate for resonant frequency degradation; $\tilde{Q}\left(t\right)$ is the covariate for Q-factor degradation; $\lambda_{1}$ and $\lambda_{2}$ are weighting coefficients, with $\lambda_{1}+\lambda_{2}=1$, reflecting the relative contribution of different failure modes to overall performance; and m and n are nonlinear exponents.

The limit state function $g\left(X,t\right)>0$ indicates that the gyroscope still retains sufficient performance margin, while $g\left(X,t\right)<0$ indicates performance failure. The $g\left(X,t\right)$ in this study is defined as a gyroscope-level performance failure criterion rather than a structural fracture criterion. In practical MEMS gyroscopes, failure is considered to occur when the device can no longer satisfy the specified performance requirements, such as allowable sensitivity loss, scale-factor error, zero-rate output drift, bandwidth degradation, mode-matching tolerance, or bias instability.

#### 3.1.1. Normalized Covariates

To unify observations from different degradation processes, parameters must be normalized and dimensionless to eliminate the multi-scale issue between the two mechanisms. This enables a more accurate capture of how both failure mechanisms influence the output of the limit state function. The normalized variables are termed covariates. This paper defines the two covariates in (8) as:

(9) $$\begin{array}{c} \begin{array}{c} \tilde{f}(t)=\frac{f_{th}-f\left(t\right)}{f_{th}}\\ \tilde{Q}\left(t\right)=\frac{\frac{1}{Q_{th}}-\frac{1}{Q\left(t\right)}}{\frac{1}{Q_{th}}} \end{array} \end{array}$$

where $f_{th}$ and $Q_{th}$ represent the resonant frequency and Q-factor degradation thresholds, respectively; and $f\left(t\right)$ and $1/Q\left(t\right)$ denote the resonant frequency and Q-factor degradation process models, respectively.

It is worth noting that $\tilde{f}(t)$ in (9) is represented by the absolute drift of a single resonant frequency. However, for a complete MEMS gyroscope, the same covariate can be defined using frequency split or mode mismatch; for example,

$$\tilde{f}{\left(t\right)}^{\prime }=\frac{\left|\Delta f(t)-\Delta f_{0}\right|}{{\Delta f}_{th}},\Delta f(t)=f_{s}(t)-f_{d}(t)$$

where $\Delta f_{0}$ is the initial or designed frequency split and ${\Delta f}_{th}$ is the allowable frequency-split variation determined by the gyroscope-level performance requirement. If the bandwidth effect is considered, a normalized mode-mismatch indicator may also be defined as

$$\tilde{f}{\left(t\right)}^{\prime \prime }=\frac{\left|f_{s}(t)-f_{d}(t)\right|}{f_{s}(t)/\left(2Q_{s}(t)\right)}$$

where $Q_{s}\left(t\right)$ is the quality factor of the sense mode. Therefore, the proposed limit state framework is not restricted to absolute resonant frequency drift; it can incorporate frequency split or mode mismatch by redefining the frequency-related covariate.

#### 3.1.2. Engineering Weighting Method Based on Scenario Expert Knowledge and Observation Data

To quantify the relative contributions of resonant frequency drift and Q-factor degradation to system failure, this section proposes an engineering weighting method based on scenario-specific expert knowledge and observational data. This approach first establishes a set of baseline weights based on domain expert knowledge and long-term engineering experience within the application scenario. These weights are then calibrated using actual observational data, ensuring the weighting coefficients possess both prior rationality and case-specific relevance while remaining adaptable to diverse engineering applications.

In this paper, the baseline weights determined by experts based on long-term engineering experience are denoted as $\lambda_{1}^{p}$ and $\lambda_{2}^{p}$.

To capture individual variations in degradation behavior under specific batches, processes, or stress conditions, calibration using observational data is required. The fundamental principle is that parameters exhibiting faster and more pronounced degradation contribute greater risk. For a set of observed degradation trajectories $\left\{f_{j}\left(t\right),Q_{j}\left(t\right)\right\}$, we calculate their average normalized degradation rate over the observation period:

(10) $$\begin{array}{c} \begin{array}{c} R_{f}=\frac{1}{M}\sum_{j=1}^{M}\frac{\left|f_{j}\left(T\right)-f_{j}\left(0\right)\right|}{f_{j}\left(0\right)T}\\ R_{Q}=\frac{1}{M}\sum_{j=1}^{M}\frac{\frac{1}{Q_{j}\left(T\right)}-\frac{1}{Q_{0}\left(0\right)}}{\frac{1}{Q_{j}\left(0\right)T}} \end{array} \end{array}$$

where $R_{f}$ and $R_{Q}$ denote the average relative rates of degradation for resonant frequency and Q-factor, respectively; T represents the duration of the observation period; $f_{j}\left(T\right)$ and $Q_{j}\left(T\right)$ denote the terminal values of resonant frequency and Q-factor degradation for the j-th degradation trajectory within the observation period; $f_{j}\left(0\right)$ and $Q_{j}\left(0\right)$ denote the initial values of resonant frequency and Q-factor for the j-th degradation trajectory; and M indicates the number of observed trajectories.

Combining expert knowledge with the average relative velocity from (10), the final weights are calculated as:

(11) $$\begin{array}{c} \begin{array}{c} \lambda_{1}=\frac{\eta \cdot \lambda_{1}^{p}+\left(1-\eta \right)\cdot \frac{R_{f}}{\left(R_{f}+R_{Q}\right)}}{\eta +\left(1-\eta \right)}\;\\ \lambda_{2}=1-\lambda_{1} \end{array} \end{array}$$

where η represents the confidence factor, which adjusts the credibility of the average relative rate derived from expert knowledge and observational data. When data are abundant, η can be set to a smaller value, allowing data to dominate the weighting. When data are scarce, a larger value can be selected, placing greater reliance on expert knowledge.

#### 3.1.3. Nonlinear Exponents

The nonlinear exponents m and n are introduced to characterize the nonlinear cumulative effects during degradation. If m=n=1, m=0,n=1, or m=1,n=0, the model represents a linear superposition of the two mechanisms, assuming degradation risk is linearly proportional to the degree of deviation from the threshold.

When m (or n) assumes other values, Equation (8) exhibits nonlinearity, indicating that the risk associated with degradation of the Q-factor (resonant frequency) evolves in a nonlinear manner. This aligns with the engineering observation that many degradation processes rapidly escalate and become nonlinear beyond a certain threshold. It is important to note that (8) represents the limit state function for the synergistic degradation of both mechanisms. An indicator function is defined based on whether it crosses the zero point to characterize failure. Therefore, both m and n can only take odd values to prevent the limit state function from failing.

### 3.2. Random Degradation Model of Mechanism-Associated Performance Parameters

#### 3.2.1. Resonant Frequency Degradation Process Model

Cumulative damage to stiffness has been confirmed to exist in MEMS silicon thin-film devices [44,45,46,47]. Although the underlying physical or chemical causes remain debated, Kahn et al. indicated that fatigue phenomena in microscale polysilicon occur independently of environmental atmosphere, even under vacuum conditions [48]. The root cause of this phenomenon lies in material fatigue of silicon microstructures under long-term cyclic stress. Its macroscopic manifestation is a quasi-monotonic decrease in effective Young’s modulus, accompanied by the intrinsic randomness of microdefect evolution. Referencing research data from Cheng et al. [21], this paper employs a Wiener process with linear drift for characterization:

(12) $$\begin{array}{c} f\left(t\right)=\mu_{f}t+\sigma_{f}B_{f}\left(t\right) \end{array}$$

where $\mu_{f}$ is the drift coefficient as a macroscopic representation of the fatigue damage rate whose absolute value directly reflects the material’s fatigue strength at a specific stress level; $\sigma_{f}$ is the diffusion coefficient, quantifying the intensity of microscopic randomness during resonant frequency degradation; and $B_{f}\left(t\right)$ represents standard Brownian motion associated with frequency-related degradation satisfying $B_{f}\left(t\right)\sim N\left(0,t\right)$, characterizing the non-predictability of fluctuations in the “next step” during degradation.

#### 3.2.2. Q-Factor Degradation Process Model

In our previous research, we established a physical Q-factor degradation trajectory to describe the performance of MEMS resonator quality factors during the initial phase. Building upon this prior work, this paper introduces an inductive process incorporating drift in the physical degradation trajectory:

(13) $$\begin{array}{c} \frac{1}{Q\left(t\right)}=\alpha_{0}-\alpha_{1}\exp\left[-\alpha_{D}^{2}t\right]+\sigma_{Q}B_{Q}\left(t\right) \end{array}$$

where $\alpha_{0}$ represents parameters related to natural constants and structural design, ensuring the Q-factor in the progressive saturation state; $\alpha_{1}$ and $\alpha_{D}$ are influenced by factors such as structure, temperature, and materials, characterizing the Q-factor degradation rate; $\sigma_{Q}$ characterizes random fluctuations beyond the physical degradation trajectory; and $B_{Q}\left(t\right)$ represents the standard Brownian motion associated with Q-factor degradation, satisfying $B_{Q}\left(t\right)\sim N\left(0,t\right)$.

### 3.3. Reliability Assessment of Synergistic Degradation by Two Mechanisms

When m=n=1, m=0,n=1, or m=1,n=0 in Equation (8), the analytical expression for the system reliability function can be derived. If m or n is neither 1 nor 0, then $g\left(X,t\right)$ becomes a nonlinear function, typically precluding closed-form analytical solutions and requiring numerical solution via Monte Carlo simulation.

#### 3.3.1. Linear Limit State

To ensure the feasibility of the analytical solution, the following two reasonable assumptions are made:

1. Where m=n=1, the limit state function is a linear combination of two normalized degradation quantities;
2. The stochastic processes shown in (12) and (13) are independent of each other.

Regarding assumption 2 mentioned above, we need to clarify that to obtain a closed-form reliability expression under the linear limit state, this subsection first adopts a baseline independence assumption. Specifically, the stochastic components of the frequency-related degradation process and Q-factor degradation process are assumed to be driven by independent Brownian motions. This assumption is used for analytical tractability and for the present reduced-order simulation case, where the two degradation paths are generated from two mechanism-specific simulations without a shared stochastic environmental input.

By the properties of the Wiener process for any fixed time t, we have $f\left(t\right)\sim N\left(\mu_{f}t,\sigma_{f}^{2}t\right)$ and $1/Q\left(t\right)\sim N\left(\alpha_{0}-\alpha_{1}e^{-\alpha_{D}^{2}t},\sigma_{Q}^{2}t\right)$. Thus, the limit state functions can be calculated separately with $g\left(X,t\right)$; at time t, these also follow a normal distribution, with the mean function $\mu_{g}$ and variance function $\sigma_{g}^{2}$ given by:

(14) $$\begin{array}{l} \mu_{g}\left(t\right)=\left|\lambda_{1}\right|\cdot \frac{f_{th}-\mu_{f}t}{f_{th}}+\left|\lambda_{2}\right|\cdot Q_{th}\left(\frac{1}{Q_{th}}-\alpha_{0}+\alpha_{1}e^{-\alpha_{D}^{2}t}\right)\\ \sigma_{g}^{2}\left(t\right)={\left|\lambda_{1}\right|}^{2}\cdot \frac{\sigma_{f}^{2}}{f_{th}^{2}}\cdot t+{\left|\lambda_{2}\right|}^{2}\cdot Q_{th}^{2}\sigma_{Q}^{2}\cdot t \end{array}$$

Therefore, the reliability function can be calculated as:

(15) $$\begin{array}{c} R\left(t\right)=\Phi \left(\frac{\left|\lambda_{1}\right|\cdot \frac{f_{th}-\mu_{f}t}{f_{th}}+\left|\lambda_{2}\right|\cdot Q_{th}\left(\frac{1}{Q_{th}}-\alpha_{0}+\alpha_{1}e^{-\alpha_{D}^{2}t}\right)}{\sqrt{t}\cdot \sqrt{\frac{{\left|\lambda_{1}\right|}^{2}\sigma_{f}^{2}}{f_{th}^{2}}+{\left|\lambda_{2}\right|}^{2}Q_{th}^{2}\sigma_{Q}^{2}}}\right) \end{array}$$

The reliability function shown in Equation (15) comprehensively reflects the synergistic effect of two degradation mechanisms under linear limit states. The deterministic drift term in the numerator dominates the long-term degradation trend, while the diffusion term in the denominator characterizes the uncertainty of random fluctuations.

For practical MEMS gyroscopes, correlated degradation can be incorporated by replacing the independent Brownian motions with correlated Brownian motions. We let $B_{f}(t)$ and $B_{Q}(t)$ denote the Brownian motions associated with frequency-related degradation and Q-factor degradation, respectively. Their correlation can be defined as:

$$Cov\left[B_{f}\left(t\right),B_{Q}\left(t\right)\right]=\rho t,-1\leq \rho \leq 1$$

where ρ=0 corresponds to the independent case used in the baseline analytical model, while ρ≠0 represents correlated degradation induced by common stresses.

For the linear limit state, the correlated case can still be handled analytically by modifying the variance term of the combined degradation index. The reliability function with correlation can be calculated as:

$$R\left(t\right)=\Phi \left(\frac{\left|\lambda_{1}\right|\cdot \frac{f_{th}-\mu_{f}t}{f_{th}}+\left|\lambda_{2}\right|\cdot Q_{th}\left(\frac{1}{Q_{th}}-\alpha_{0}+\alpha_{1}e^{-\alpha_{D}^{2}t}\right)}{\sqrt{t}\cdot \sqrt{\frac{{\left|\lambda_{1}\right|}^{2}\sigma_{f}^{2}}{f_{th}^{2}}+{\left|\lambda_{2}\right|}^{2}Q_{th}^{2}\sigma_{Q}^{2}+2\rho \frac{\left|\lambda_{1}\right|}{f_{th}}\left|\lambda_{2}\right|Q_{th}\sigma_{f}\sigma_{Q}}}\right)$$

When ρ=0, this expression reduces to the independent case. Therefore, the proposed framework can include correlated degradation processes without changing the basic limit state formulation.

#### 3.3.2. Nonlinear Limit State

When the generalized limit state function $g\left(X,t\right)$ exhibits nonlinearity (m or n is neither 1 nor 0), the system reliability $R\left(t\right)=P\left(g\left(X,t\right)>0\right)$ cannot be obtained through a closed-form analytical solution. Due to its universality regarding problem dimensions and nonlinear forms, the Monte Carlo (MC) method becomes the fundamental approach for solving such problems. Its core principle involves statistically estimating the frequency of safe system operation through extensive random sampling, thereby providing an unbiased estimate of the reliability function.

This paper employs the MC method to simulate synergistic degradation processes. For a given time t, the joint distribution of the random state vector ${\left[f\left(t\right),Q^{-1}\left(t\right)\right]}^{T}$ is entirely determined by (12) and (13). The specific implementation process is as follows:

**Step 1:** Sample generation. We conduct N independent random trials. In the i-th trial, we generate a random state sample pair following the model distribution based on the distribution properties of the Wiener process at a fixed time:

(16) $$\begin{array}{c} \begin{array}{c} f^{\left(i\right)}\left(t\right)=\mu_{f}t+\sigma_{f}\sqrt{t}\cdot Z_{f}^{\left(i\right)}\\ {\left[Q^{\left(i\right)}\left(t\right)\right]}^{-1}=\alpha_{0}-\alpha_{1}\exp\left(-\alpha_{D}^{2}t\right)+\sigma_{Q}\sqrt{t}\cdot Z_{Q}^{\left(i\right)} \end{array} \end{array}$$

where $Z_{f}^{\left(i\right)},Z_{Q}^{\left(i\right)}\overset{i.i.d.}{\sim }N(0,1)$ represents standard normal random numbers, while $\overset{i.i.d.}{\sim }$ denotes independent and identically distributed random variables.

**Step 2:** State mapping and failure criterion evaluation. We map each random sample $\left(f^{\left(i\right)}(t),{\left[Q^{\left(i\right)}(t)\right]}^{-1}\right)$ to a normalized performance metric and compute the limit state function value:

(17) $$\begin{array}{c} \begin{array}{c} {\tilde{f}}^{\left(i\right)}\left(t\right)=\frac{f_{th}-f^{\left(i\right)}\left(t\right)}{f_{th}}\\ {\tilde{Q}}^{\left(i\right)}\left(t\right)=Q_{th}\left(Q_{th}^{-1}-{\left[Q^{\left(i\right)}\left(t\right)\right]}^{-1}\right)\\ g^{\left(i\right)}\left(X,t\right)=\left|\lambda_{1}\right|\cdot {\left[{\tilde{f}}^{\left(i\right)}\left(t\right)\right]}^{m}+\left|\lambda_{2}\right|\cdot {\left[{\tilde{Q}}^{\left(i\right)}\left(t\right)\right]}^{n} \end{array} \end{array}$$

**Step 3:** Statistical inference. We first define the indicator function $I^{\left(i\right)}$ to indicate whether the sample causes system failure:

(18) $$\begin{array}{c} I^{\left(i\right)}=\left\{\begin{array}{c} 1,\mathrm{if}\;g^{\left(i\right)}\left(X,t\right)\leq 0\\ 0,\mathrm{if}{\;g}^{\left(i\right)}\left(X,t\right)>0 \end{array}\right. \end{array}$$

where $I^{\left(i\right)}=1$ denotes system failure, while $I^{\left(i\right)}=0$ indicates system safety. Thus, the reliability of the system at time t can be unbiasedly estimated by the sample mean of the indicator function:

(19) $$\begin{array}{c} \hat{R}\left(t\right)=1-\hat{p}\left(t\right)=1-\frac{1}{N}\sum_{i=1}^{N}I^{\left(i\right)} \end{array}$$

**Step 4:** Error analysis. Since the Monte Carlo estimator is a statistic, assessing its precision is critical. The indicator function $I^{\left(i\right)}$ follows a Bernoulli distribution. Using the Central Limit Theorem, when the sample size is sufficiently large, $\hat{p}\left(t\right)$ approximates a normal distribution. Based on this, a $\left(1-\alpha \right)\times 100\%$ confidence interval for the system reliability estimate $\hat{R}\left(t\right)$ can be constructed:

(20) $$\begin{array}{c} \hat{R}\left(t\right)\in \left[1-\hat{p}-z_{1-\frac{\alpha }{2}}\sqrt{\frac{\hat{p}\left(1-\hat{p}\right)}{N}},1-\hat{p}+z_{1-\frac{\alpha }{2}}\sqrt{\frac{\hat{p}\left(1-\hat{p}\right)}{N}}\right] \end{array}$$

where $z_{1-\alpha /2}$ represents the $\left(1-\alpha /2\right)$ quantile of the standard normal distribution. Equation (20) quantifies the statistical uncertainty of the simulation results, providing a clear measure of their reliability.

## 4. Parameter Estimation

To identify model parameters from observed degradation data, this section derives maximum likelihood estimators for both the linear random degradation model of resonant frequency and the nonlinear random degradation model of Q-factor. Corresponding likelihood function optimization algorithms are provided, establishing a robust parameter foundation for subsequent reliability analysis.

As analyzed earlier, the parameters requiring estimation in the degradation and reliability models are $\Theta =\left(\mu_{f},\sigma_{f},\alpha_{0},\alpha_{1},\alpha_{D},\sigma_{Q}\right)$. For analytical convenience, we denote this parameter vector as $\Theta =\left(\theta_{f},\theta_{Q}\right)$, where $\theta_{f}=\left(\mu_{f},\sigma_{f}\right)$ and $\theta_{Q}=\left(\alpha_{0},\alpha_{1},\alpha_{D},\sigma_{Q}\right)$. Based on the meanings of each parameter, it can be understood that $\theta_{f}$ represents the degradation characteristics of the resonant frequency drift degradation process $f\left(t\right)$, while $\theta_{Q}$ represents the degradation characteristics of the Q-factor degradation process. This section details a maximum likelihood estimation-based parameter estimation framework. This method fully utilizes degradation data from all time points, simultaneously estimates deterministic drift parameters and stochastic diffusion parameters, and provides good statistical properties.

Suppose degradation experiments are conducted on M identical devices. From the time series $t_{1}<t_{2}<\ldots <t_{n}$, the measured resonant frequency degradation sequence $\left\{f_{m,i}\right\}$ and reciprocal Q-factor sequence $\left\{y_{m,i}\right\}$ are obtained, where m denotes the device number, i represents the observation time point index, and N indicates the sequence length.

### 4.1. Parameter Estimation for Resonant Frequency Degradation Process Model

Although the resonant frequency degradation model (8) established in this paper follows a Wiener process with linear drift, under the maximum likelihood estimation (MLE) framework for Brownian motion with independent increments, the estimation of the drift term ultimately relies solely on the initial and final observation values, completely ignoring intermediate path information. In practical applications, if the initial and final points happen to be affected by noise or outliers, the estimation results will be highly unstable. Furthermore, the violation of the homoscedasticity assumption in the Ordinary Least Squares (OLS) method imposes limitations on its application. To address this, this paper defines the state space using time intervals and employs recursive parameter estimation via Kalman filtering. This approach fully leverages the contribution of intermediate data to achieve more accurate parameter estimates.

First, we consider the j-th observation among M observations. Based on the increment independence of the Wiener process, we consider the time interval $∆t_{i}=t_{i}-t_{i-1},i=2,3,\ldots ,N$ as follows:

(21) $$\begin{array}{c} f\left(t_{i}\right)=f\left(t_{i-1}\right)+\mu_{f}∆t_{i}+\delta_{i},{\;\delta }_{i}\sim N\left(0,\sigma_{f}^{2}∆t_{i}\right) \end{array}$$

Simultaneously, we assume the observation contains measurement error ε and that $\varepsilon \sim N\left(0,\sigma_{\varepsilon }^{2}\right)$. Therefore, when estimating model parameters using observations, we also incorporate the measurement error ε. We let the vector of parameters to be estimated be denoted as $\theta_{1}={\left[\mu_{f},\sigma_{f}^{2},\sigma_{\varepsilon }^{2}\right]}^{T}$. Using the Kalman filter recursive process, we define the negative log-likelihood function for the j-th observation:

(22) $$\begin{array}{c} l_{i}\left(\theta_{f}\right)=-\frac{1}{2}\sum_{i=2}^{N}\left(log\left(2\pi \right)+log\left|S_{j,i}\right|+\eta_{j,i}^{2}S_{j,i}^{-1}\right) \end{array}$$

where $\eta_{j,i}=k_{j,i}-{\hat{k}}_{j,i|i-1}$ denotes the residual between the i-th observation $f_{j,i}$ and the state ${\hat{f}}_{j,i|i-1}$ predicted based on the observation $f_{j,1},\ldots ,f_{j,i-1}$; and $S_{j,i}=P_{j,i|i-1}+\sigma_{\varepsilon }^{2}$ represents the residual covariance, jointly formed by the prediction covariance $P_{j,i|i-1}$ and the variance of the measurement error $\sigma_{\varepsilon }^{2}$.

Since the M observations are independent, the total negative log-likelihood function is the sum of the negative log-likelihood functions for each observation, namely:

(23) $$\begin{array}{c} l_{total}\left(\theta_{f}\right)=-\frac{1}{2}\sum_{j=1}^{M}\sum_{i=2}^{N}\left[\log\left(2\pi \right)+logS_{j,i}+\frac{\eta_{j,i}^{2}}{S_{j,i}}\right] \end{array}$$

The Gauss–Newton method employed in this paper minimizes the negative log-likelihood function (23) to estimate parameters of the resonant frequency degradation model. The algorithmic framework utilizing Kalman filter recursive estimation and Gauss–Newton method for updating stiffness degradation model parameters is illustrated in Algorithm 1.
**Algorithm 1**: Kalman Filter Recursive Estimation and Gauss–Newton Method for Updating Parameters of the Resonant Frequency Degradation Model**Input:**Observation sequence ${\left\{t_{i},f_{i}\right\}}_{M,N}$//$t_{i}$: Observation time, $f_{i}$: Observed degradation values, M: Number of observed objects, N: Observation sequence length
Initial Parameters: $\theta_{f}^{\left(0\right)}={\left[\mu_{f}^{\left(0\right)},log\left(\sigma_{f}^{2\left(0\right)}\right),log\left(\sigma_{\varepsilon }^{2\left(0\right)}\right)\right]}^{T}$
Convergence Threshold: ϵ
**Output:**Estimated parameters: $\theta_{f}={\left[\mu_{f},\sigma_{f}^{2},\sigma_{\varepsilon }^{2}\right]}^{T}$1**:**  Calculation interval: $∆t_{i}=t_{i}-t_{i-1},i=2,3,\ldots ,N$2**:**  Initialize optimizer: k=0 // Iteration count3**:**  do:4**:**   // Calculate total negative log-likelihood function for Kalman filter5**:**    $\left[nll\left(\theta_{f}^{k}\right)\right]=KalmanFilter\left(\theta_{f}^{k},\left\{\begin{array}{c} t_{i},f_{i} \end{array}\right\},∆t\right)$6**:**     // Gauss-Newton method for parameter update7**:**    $\theta_{f}^{k+1}=\theta_{f}^{k}-{\left(H^{k}\right)}^{-1}\nabla nll\left(\theta_{f}^{k}\right)$8**:**    k=k+19**:**  until $\left\|\theta_{f}^{k}-\theta_{f}^{k-1}\right\|<\epsilon$10**:**   // Extract final parameters11**:**  ${\hat{\mu }}_{f}=\theta_{f}^{k}\left(1\right)$12**:**  ${\hat{\sigma }}_{f}^{2}=exp\left(\theta_{f}^{k}\left(2\right)\right)$  // Exponential transformation to ensure positive definiteness13**:**  ${\hat{\sigma }}_{\varepsilon }^{2}=exp\left(\theta_{f}^{k}\left(3\right)\right)$14**:**   Return: ${\hat{\theta }}_{f}=\left[{\hat{\mu }}_{f},{\hat{\sigma }}_{f}^{2},{\hat{\sigma }}_{\varepsilon }^{2}\right]$

As evidenced, the estimation of $\mu_{f}$ relies on all observations, suppressing the influence of outliers at the beginning and end. This paper establishes a unified framework for estimating parameters in the resonant frequency degradation model. The estimation method can be extended to time-varying drift rate models or stochastic diffusion rate models, making it applicable to a wider range of scenarios.

### 4.2. Parameter Estimation for the Q-Degradation Process Model

The Q-factor degradation model established in this paper exhibits high nonlinearity. Therefore, parameter estimation for this model still originates from the increment independence of the Wiener process. Based on the increment distribution, a log-likelihood function is constructed, and maximum likelihood estimation is performed through numerical optimization strategies to ultimately obtain the optimal model estimates. The specific steps are as follows.

First, we consider the j-th observation among M observations. For the time interval $∆t_{i}=t_{i}-t_{i-1},i=2,3,\ldots ,N$, the incremental model for Q-factor degradation is:

(24) $$\begin{array}{c} ∆Q_{i}=\frac{1}{Q\left(t_{i}\right)}-\frac{1}{Q\left(t_{i-1}\right)}=\alpha_{1}\left[exp\left(-\alpha_{D}^{2}t_{i-1}\right)-exp\left(-\alpha_{D}^{2}t_{i}\right)\right]+\sigma_{Q}∆B,i=2,\ldots ,N \end{array}$$

where $∆B=B\left(t_{i}\right)-B\left(t_{i-1}\right)$ represents the increment of Brownian motion. Based on the Markov property of Brownian motion and observational data (sequence of reciprocal Q values $\left\{y_{m,i}\right\}$), we obtain:

(25) $$\begin{array}{l} y_{j,1}\sim N\left(\alpha_{0}-\alpha_{1}e^{-\alpha_{D}^{2}t},\sigma_{Q}^{2}t_{1}\right)\;\\ y_{j,i}|y_{j,i-1}\sim N\left(y_{j,i-1}+\alpha_{1}\left[exp\left(-\alpha_{D}^{2}t_{i-1}\right)-exp\left(-\alpha_{D}^{2}t_{i}\right)\right],\sigma_{Q}^{2}\left(t_{i}-t_{i-1}\right)\right) \end{array}\;$$

Therefore, the log-likelihood function for the j-th observation is:

(26) $$\begin{array}{c} l_{j}\left(\theta_{Q}\right)=-\frac{1}{2}log\left(2\pi \sigma_{Q}^{2}t_{1}\right)-\frac{{\left(D_{j,1}-∆f_{1}\right)}^{2}}{2\sigma_{Q}^{2}t_{1}}-\frac{1}{2}\sum_{i=2}^{N}\left[log\left(2\pi \sigma_{Q}^{2}{∆}_{i}\right)+\frac{{\left(D_{j,i}-∆f_{i}\right)}^{2}}{\sigma_{Q}^{2}{∆}_{i}}\right] \end{array}$$

Since the observation curves are mutually independent, the total log-likelihood function is the sum of the log-likelihoods for each observation:

(27) $$\begin{array}{c} l_{total}\left(\theta_{Q}\right)=\sum_{j=1}^{M}l_{j}\left(\theta_{Q}\right)=-\frac{M}{2}\sum_{i=1}^{N}log\left(2\pi \sigma_{Q}^{2}{∆}_{t_{i}}\right)-\frac{1}{2\sigma_{Q}^{2}}\sum_{i=1}^{N}\frac{S_{i}}{∆t_{i}} \end{array}$$

where $S_{i}=\sum_{j=1}^{M}{\left(y_{j,i}-y_{j,i-1}-\alpha_{1}\left[exp\left(-\alpha_{D}^{2}t_{i-1}\right)-exp\left(-\alpha_{D}^{2}t_{i}\right)\right]\right)}^{2},i=1,2,\ldots ,N$.

By maximizing (27), we obtain the optimal estimate. We simplify this using a nonlinear mixed-effects model. By fixing parameters $\left(\alpha_{0},\alpha_{1},\alpha_{D}\right)$ and setting $\partial l_{total}\left(\theta_{Q}\right)/\partial \sigma_{Q}^{2}=0$, we solve for the result and substitute it back into (27) to obtain the contour likelihood function:

(28) $$\begin{array}{c} l_{p}\left(\theta \right)=-\frac{MN}{2}\log\left(\sum_{i=1}^{N}\frac{S_{i}}{{∆}_{i}}\right) \end{array}$$

Therefore, maximizing $l_{total}\left(\theta_{Q}\right)$ is equivalent to minimizing the following objective function:

(29) $$\begin{array}{c} S_{total}\left(\theta_{Q}\right)=\sum_{i=1}^{N}\frac{1}{∆t_{i}}\sum_{j=1}^{M}{\left(D_{j,i}-∆f_{i}\right)}^{2} \end{array}$$

The optimization process for objective function (29) employs the same Gauss–Newton method as in Section 4.1, which is not repeated here.

## 5. Case Study

### 5.1. Data Acquisition

Before presenting the two degradation datasets, the simulation-based data acquisition procedure is briefly described. In this study, the degradation data used for method verification are obtained from simplified multiphysics simulations. Specifically, representative MEMS structural models are first established for the two degradation channels. Then, degradation-related physical conditions are progressively varied in the simulations to obtain the corresponding changes in resonant frequency and Q-factor. The simulated outputs are fitted to obtain the nominal physical degradation trajectories and the corresponding model parameters. Finally, stochastic degradation trajectories are generated by introducing random-process variations around the fitted physical trajectories, so that the simulated data can reflect the randomness of practical degradation processes. The generated datasets are subsequently used for parameter estimation and reliability modeling in the following sections.

In this case study, the term “double-ended fixed beam” is used consistently for the frequency-related degradation submodel, whereas the term “cantilevered MEMS mass block” is used consistently for the Q-factor degradation submodel. These two structures represent two mechanism-specific reduced-order models corresponding to different degradation channels in the proposed reliability framework.

#### 5.1.1. Resonant Frequency Degradation Simulation

**Figure 2:**
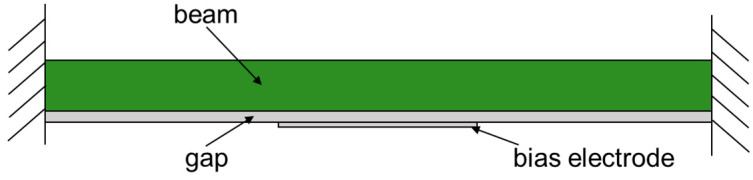
Geometric model of the double-ended fixed beam used as the frequency-related degradation submodel. The beam, air gap, and bias electrode are included in the multiphysics simulation.

As previously mentioned, one significant cause of resonant frequency drift is fatigue damage in silicon material under alternating loads. Cheng et al. [21] noted that the effective Young’s modulus can be used to relate MEMS resonant frequency to fatigue damage, establishing the cumulative damage formula shown in (30):

(30) $$\begin{array}{l} D=\left(C_{0}∆t^{\zeta }+1\right)\left(1-e^{-A(∆\sigma {)}^{\alpha }N^{*}}\right)\\ E=E_{0}\left(1-D\right) \end{array}$$

where ζ, $C_{0}$, α, and A represent model parameters; E denotes the effective Young’s modulus; $E_{0}$ represents the initial Young’s modulus; and D is the cumulative damage variable. This paper employs COMSOL Multiphysics (V6.2) to establish a model of a typical double-ended fixed beam in a MEMS gyroscope. The model incorporates the beam, air gap (present between the beam bottom and substrate), and bias electrodes. Its basic structure is shown in Figure 2. Key simulation parameters are listed in Table 1.

**Figure 3:**
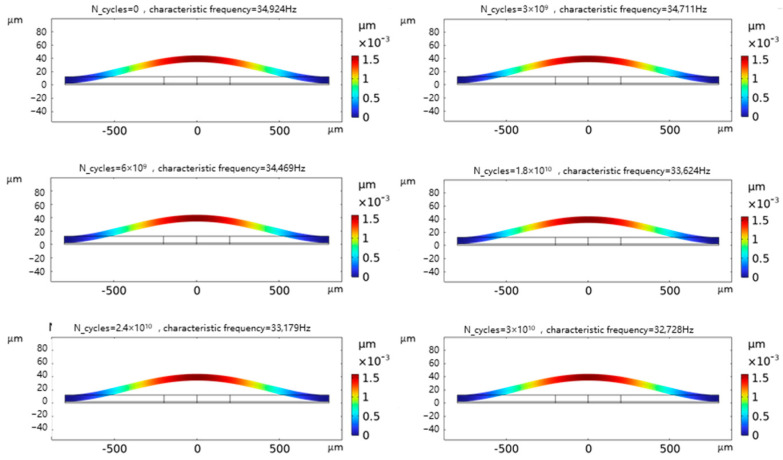
Y-direction displacement distribution and first-order natural frequency of the double-ended fixed beam under different fatigue cycle numbers. The results show the influence of stiffness degradation on the modal response and resonant frequency.

**Figure 4:**
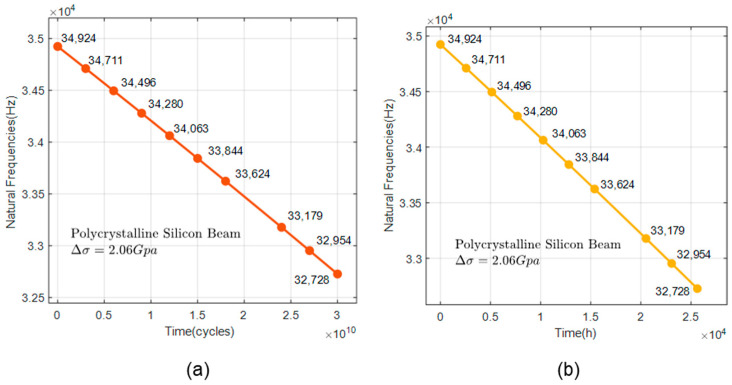
Simulated first-order natural frequency degradation of the double-ended fixed beam. (**a**) Natural frequency variation with fatigue cycle number; (**b**) natural frequency variation with operating duration.

Considering Young’s modulus damage in Equation (30), we analyzed the Y-direction displacement and first-order natural frequency of double-ended fixed beam model at different cycle counts. The results are shown in Figure 3. Consistent with our expectations, the maximum displacement occurs at the drive electrode section, and the first-order natural frequency exhibits a pronounced decreasing trend. The variation in the natural frequency with cyclic time (number of cycles) is shown in Figure 4. Its degradation exhibits a pronounced linear characteristic within the observed time domain, consistent with experimental results by Muhlstein [44,45,46].

**Figure 5:**
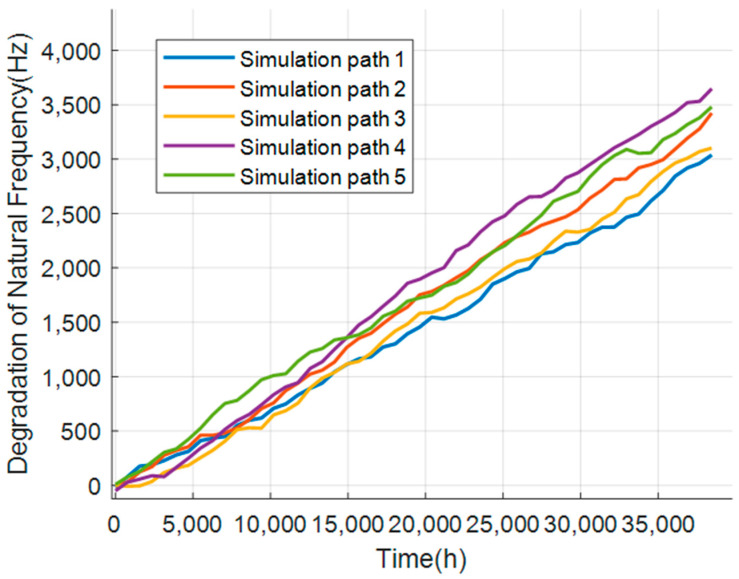
Five stochastic resonant frequency degradation trajectories generated from the fitted linear drift model and Wiener process random variations. These trajectories are used as the frequency-related degradation dataset for parameter estimation and reliability analysis.

Simulated data are idealized, whereas actual degradation processes are inherently stochastic. A linear regression is first performed on the simulated degradation data, resulting in a slope of $8.57\times 10^{-2}$. The parameters $\mu_{f}=8.57\times 10^{-2}$ and $\sigma_{f}=1.2$ in Equation (12) are then specified, and five random degradation trajectories are generated, as illustrated in Figure 5. The time scale is expanded to $4\times 10^{4}$ h to facilitate observation of failure times under specified thresholds.

#### 5.1.2. Q-Factor Degradation Simulation

**Figure 6:**
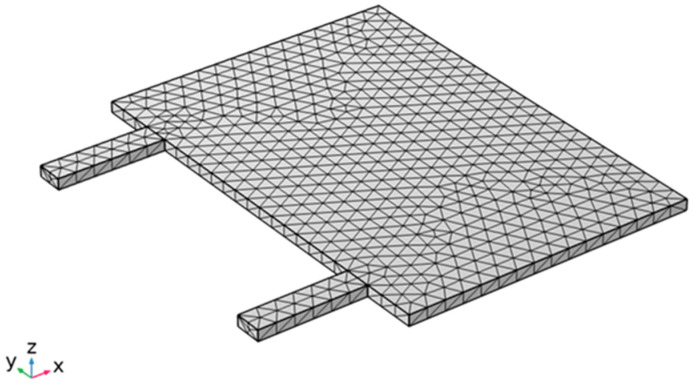
Free tetrahedral mesh of the cantilevered MEMS mass block used as the damping-related Q-factor degradation submodel. The model is used to simulate squeeze-film damping and pressure-related Q-factor variation.

In our previous research, the sealing degradation process of vacuum-encapsulated MEMS was divided into two stages, with the first stage establishing a physical degradation trajectory of the Q-factor based on long-term observations. This is because the Q-factor serves as the key parameter describing energy loss in resonators and is highly sensitive to damping changes. When the encapsulation pressure is below 1 mtorr, changes in the Q-factor are dominated by air damping [49]. Air damping comprises squeeze-film damping and sliding-film damping, with squeeze-film damping being the primary energy loss mechanism in MEMS devices [50]. As the overlap area increases, the squeeze-film damping effect becomes more pronounced. Therefore, a cantilevered MEMS mass block model was established in COMSOL Multiphysics to simulate squeeze-film damping and the corresponding Q-factor variations under progressively increasing air pressure. Figure 6 illustrates the meshed cantilevered MEMS mass block model. In this reduced-order cantilevered MEMS mass block model, the two cantilever beams are attached to a double-ended fixed beam or an elastic support beam, whereas no fixed constraints are applied to the remaining surfaces. The key geometric parameters of the model are summarized in Table 2.

**Figure 7:**
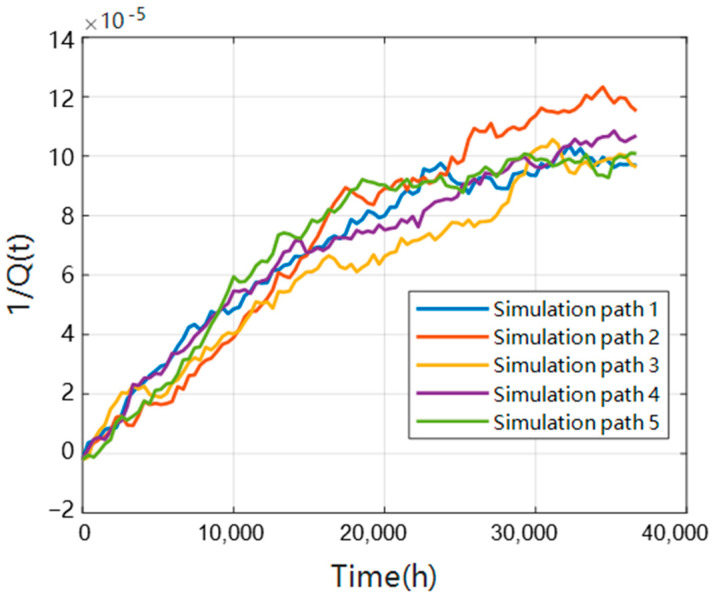
Five stochastic reciprocal Q-factor degradation trajectories generated from the fitted physical degradation trajectory and Wiener process random variations. These trajectories are used as the damping-related degradation dataset for parameter estimation and reliability analysis.

To obtain stochastic degradation data based on the simulation results, the same strategy as that described in Section 5.1.1 is adopted. According to the least-squares fitting results of the physical degradation trajectory, the parameters in (13) are set as $\alpha_{0}=14.48\times 10^{-5}$; $\alpha_{1}=20.04\times 10^{-5}$; $\alpha_{D}=5.91\times 10^{-3}$; and $\sigma_{Q}=1.00\times 10^{-7}$. Subsequently, five stochastic Q-factor degradation trajectories are randomly generated, as shown in Figure 7.

### 5.2. Parameter Estimation Results

Parameter estimation was performed on the resonant frequency degradation simulation data using the parameter estimation method proposed in Section 4.1. The results are shown in Table 3.

**Figure 8:**
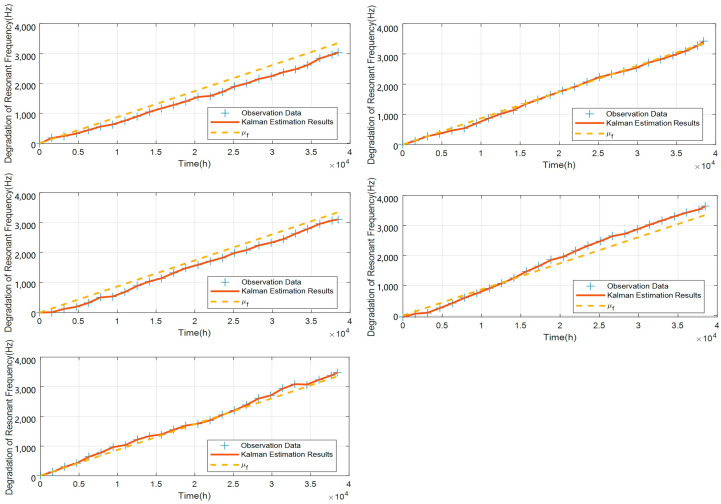
Kalman filter-based estimation results for the simulated resonant frequency degradation trajectories. The estimated trajectories are compared with the simulated observation data to evaluate the parameter estimation performance.

Analysis of Table 3 reveals that for both joint estimation of multiple datasets and average independent estimation, the estimated linear drift coefficient remains within 2% of the preset parameter. However, for the diffusion coefficient $\sigma_{f}^{2}$, the joint estimation result (difference of 5.83%) significantly outperforms the average independent estimation (difference of 16.7%). We attribute this discrepancy to the number of simulation samples. We also compare Kalman filter estimates with observational data for trajectories with varying degradation patterns in Figure 8.

**Figure 9:**
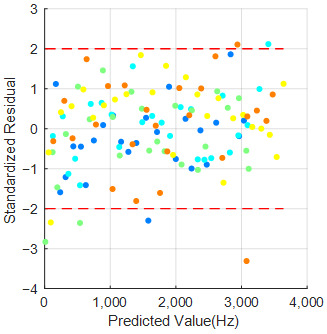
Standardized residuals of the resonant frequency degradation model at different predicted values (The colored dots denote the normalized residuals, and the two orange dashed lines correspond to the ±1.96 reference lines). The residual distribution is used to check the adequacy of the Brownian motion variance setting.

Despite errors and drift, Kalman filtering consistently predicts observed values with high accuracy. Figure 9 shows the standardized residuals fluctuating randomly within the range [−1.96, 1.96]. After normalization via ${\hat{\sigma }}_{f}\sqrt{t}$, the standardized residuals exhibit no significant heteroscedasticity, supporting the variance setting of Brownian motion in the Wiener process. This indicates the model successfully captures the time-varying variance characteristics.

**Figure 10:**
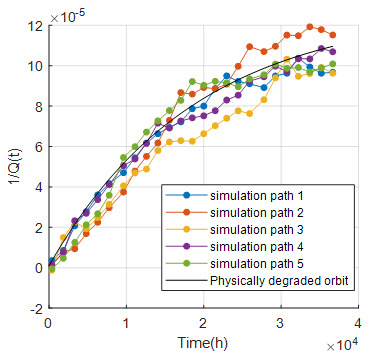
Estimated Q-factor-related degradation trajectories compared with the simulated observation data. The black solid line represents the fitted physical degradation trajectory, and the colored curves represent stochastic degradation paths.

Parameter estimation for Q-factor degraded data was performed using the method proposed in Section 4.2. The results are shown in Table 4. It can be seen that despite the nonlinearity exhibited by the Q-factor degraded physical trajectory, the differences between the estimated values and preset values are small, indicating good estimation results. The tracking estimation results for different Q-factor degraded trajectories and their comparison with observational data are displayed in Figure 10. The black solid line represents the Q-factor physical degradation trajectory under the preset parameters.

### 5.3. MEMS Reliability Analysis with Synergistic Degradation of Resonant Frequency and Q-Factor

In this section, we conduct reliability analysis using simulated degradation data and parameter estimation results for resonant frequency and Q-factor to demonstrate the feasibility of the proposed method.

#### 5.3.1. Linear Limit State Reliability Analysis and Sensitivity

When the nonlinear exponent is m=n=1, the reliability function for the MEMS gyroscope with co-degrading resonant frequency and Q-factor proposed in this paper can be analytically expressed by (14) and (15). The weighting coefficients $\lambda_{1}$ and $\lambda_{2}$ are jointly determined by expert knowledge and observational data. Considering the rapid progression of Q-factor degradation in the first stage, this paper assumes expert reference weights $\lambda_{1}^{p}=0.4$ and $\lambda_{2}^{p}=0.6$. Based on (11), both the resonant frequency and Q-factor degradation data have only five samples; thus, the confidence factor is set as η=0.7. Finally, combining the observational data with the average normalized degradation rate over one cycle yields the weighting coefficients:

(31) $$\begin{array}{c} \begin{array}{c} \lambda_{1}=0.58\\ \lambda_{2}=0.42 \end{array} \end{array}$$

**Figure 11:**
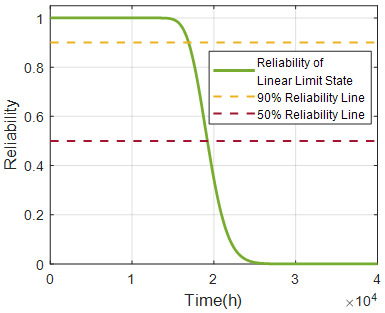
Time-varying reliability under the linear limit state using the proposed dual-indicator reliability model, with parameters set as $\lambda_{1}^{p}=0.4$, $\lambda_{2}^{p}=0.6$, and η=0.7. The curve integrates the normalized resonant frequency degradation and Q-factor degradation through the calibrated weighting coefficients.

**Figure 12:**
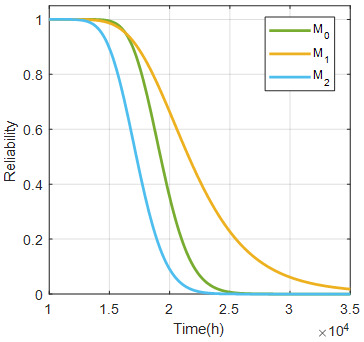
Reliability comparison between the proposed dual-indicator model and two single-indicator models: M0 denotes the proposed model combining resonant frequency degradation and Q-factor degradation, M1 denotes the Q-factor-only model, and M2 denotes the resonant frequency-only model.

The reliability function under linear limit state is shown in Figure 11, where reliability decreases to approximately 90% at $1.65\times 10^{4}$ h. Additionally, we compared the reliability between synergistic degradation of both mechanisms and degradation of a single mechanism as shown in Figure 12. The linear limit state reliability model in this paper is denoted as M_0_, the reliability model with degradation of Q-factor only is denoted as M_1_, and the reliability model with degradation of resonant frequency only is denoted as M_2_. The results in Figure 12 indicate that compared to the proposed dual-mechanism synergistic degradation reliability model, M_1_ (Q-factor degradation only) may yield overly conservative reliability analysis results, posing operational risks to equipment. Conversely, M_2_ (resonant frequency degradation only) may produce excessively aggressive reliability analysis outcomes, leading to premature shutdowns or replacements and resulting in resource wastage.

**Figure 13:**
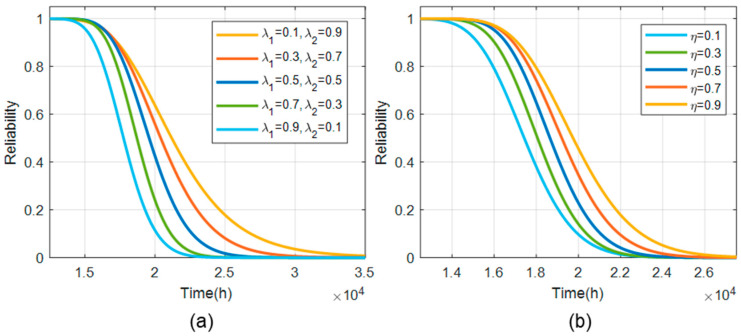
Sensitivity analysis of the linear limit state reliability function: (**a**) effect of varying the weighting coefficients of the resonant frequency and Q-factor degradation indicators; (**b**) effect of varying the expert knowledge confidence factor in the engineering weighting method.

We also analyzed the sensitivity of reliability assessments under linear limit states, as shown in Figure 13. As indicated in Figure 13a, when $\lambda_{1}$ increases (the resonant frequency contributes more to synergistic degradation), the reliability assessment becomes more aggressive. Conversely, when $\lambda_{2}$ increases (the Q-factor contributes more to synergistic degradation), the evaluation becomes more conservative. Figure 13b further analyzes the impact of the trust factor η on reliability. As η increases (indicating higher trust in expert knowledge), the reliability assessment becomes more conservative; conversely, the results become relatively aggressive.

#### 5.3.2. Nonlinear Limit State Reliability Analysis and Sensitivity

When m or n in (8) is neither 1 nor 0, the limit state function exhibits nonlinearity, making it difficult to obtain an analytical solution for the reliability function. Therefore, this study employs the Monte Carlo method described in Section 3.3.2 to estimate the reliability function under different nonlinear exponent combinations. For each time point, $N_{MC}=10^{5}$ independent degradation samples are generated from the estimated stochastic degradation models and substituted into the nonlinear limit state function. The reliability is estimated as the proportion of samples satisfying the safe condition. A fixed random seed of 42 is used to ensure reproducibility. The convergence of the Monte Carlo estimate is checked by comparing the reliability curves obtained with $N_{MC}$ and $N_{MC}/2$. The simulation is considered converged when the maximum absolute difference between the two reliability curves over the entire time grid is less than 10^−3^. The 95% confidence interval is calculated based on the Bernoulli variance of the reliability estimator as

$$\hat{R}(t)\pm 1.96\sqrt{\frac{\hat{R}\left(t\right)[1-\hat{R}(t)]}{N_{MC}}}$$

**Figure 14:**
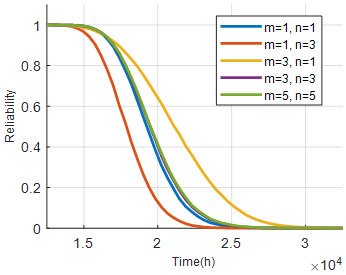
Reliability curves under nonlinear limit states with different nonlinear exponent combinations. The curves show how the nonlinear exponents m and n affect the time-varying reliability of the dual-indicator degradation model.

**Figure 15:**
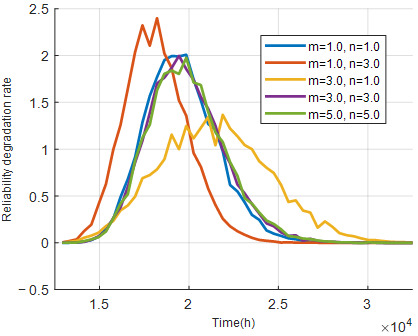
Reliability degradation-rate curves under nonlinear limit states with different nonlinear exponent combinations. The results illustrate how the nonlinear exponents affect the speed and concentration of reliability decline over time.

The results are shown in Figure 14 and Figure 15, respectively. The results indicate that, when m remains constant, increasing n shifts the reliability curve to the left and yields more aggressive reliability analysis results, all while producing a taller, slimmer reliability degradation rate curve. Conversely, when n remains constant, increasing m shifts the reliability curve to the right and yields more conservative reliability analysis results, all while producing a shorter, wider reliability degradation rate curve. When m=3,n=3 and m=5,n=5, their reliability curves (Figure 14) and reliability degradation rate curves (Figure 15) nearly coincide with those for m=1,n=1, exhibiting degradation characteristics nearly identical to the linear superposition model. This indicates that when nonlinear exponents take the same value, reliability analysis results demonstrate generalized linear properties.

## 6. Discussion

For a complete MEMS gyroscope, frequency split and mode mismatch are more directly related to gyroscope performance than the absolute drift of a single resonant frequency. The Coriolis force generated by the drive motion excites the sense mode, and the sense-mode response is governed by the transfer function near the drive frequency. Therefore, when the drive and sense resonant frequencies drift apart, the sense-mode gain decreases, leading to sensitivity loss. Since the scale factor is determined by the conversion gain from angular rate to output signal, variations in mode mismatch may also result in scale-factor drift or instability. In addition, mode mismatch may interact with quadrature coupling, demodulation phase error, temperature-induced modal drift, and closed-loop mode-matching control, thereby contributing to zero-rate output drift and bias instability. From this perspective, the proposed framework can be extended to a complete gyroscope architecture by extracting degradation indicators from the coupled drive–sense system. Specifically, the frequency-related indicator can be defined using drive–sense frequency split or mode-mismatch evolution, while the damping-related indicator can be defined using the drive- and sense-mode Q-factors or their mismatch.

Several limitations should be noted. First, the present validation is based on reduced-order multiphysics simulation data rather than long-term experimental data from packaged MEMS gyroscopes. Therefore, the reliability curves obtained in this study should be regarded as a simulation-based demonstration of the proposed reliability fusion method rather than as fully validated reliability predictions for a specific practical gyroscope. Second, the case study uses two representative reduced-order structures to extract mechanism-specific degradation indicators: the double-ended fixed beam is used as a reduced-order model for frequency-related degradation caused by stiffness degradation; and the cantilevered MEMS mass block is used as a reduced-order model for Q-factor degradation caused by damping degradation. For a specific gyroscope design, both indicators should ideally be extracted from the same full-device finite-element model, digital twin, or paired degradation experiment. Third, the baseline analytical model adopts an independence assumption between the two degradation processes. This assumption is used for analytical tractability, but practical MEMS gyroscopes may exhibit correlated degradation under common stress factors, such as temperature cycling, packaging degradation, residual stress relaxation, and aging. Finally, the weighting coefficients include expert prior information and may therefore be application dependent. Future work will focus on full-device modeling, paired experimental degradation testing, data-driven weight calibration, correlated degradation modeling, and experimental validation using long-term performance data from packaged MEMS gyroscopes.

## 7. Conclusions

This paper proposed a physics-informed dual-indicator reliability assessment framework for MEMS gyroscopes. Frequency-related degradation and Q-factor degradation were modeled using differentiated Wiener processes and fused through a generalized weighted limit state function. The limit state was interpreted as a gyroscope-level performance failure criterion, and both closed-form reliability expressions for linear limit states and Monte Carlo solutions for nonlinear limit states were provided. Reduced-order multiphysics simulation cases were used to demonstrate the proposed mechanism-to-indicator-to-reliability modeling procedure. The results show that the dual-indicator framework provides a more balanced reliability assessment than single-indicator analysis under the simulation setting and can help avoid overly conservative or aggressive reliability judgments caused by relying on a single degradation indicator. Future work will further validate the proposed framework using full-device gyroscope models and long-term experimental degradation data from packaged MEMS gyroscopes.

## Figures and Tables

**Table 1:** Key parameters of the double-ended fixed beam simulation model.

Name	Symbol	Value [μm]
Beam Length	$l_{b}$	800
Beam Thickness	$t_{b}$	10
Electrode Length	$l_{e}$	200
Air Gap Width	$w_{gap}$	2
DC Bias Voltage	V	5 [V]

**Table 2:** Key geometric parameters of cantilevered MEMS mass block model.

Name	Symbol	Value [μm]
Mass Block Length	$l_{m}$	1080
Mass Block Width	$w_{m}$	1560
Mass Block Height	$h_{m}$	40
Cantilever Length	$l_{cb}$	400
Cantilever Width	$w_{cb}$	100
Cantilever Height	$h_{cb}$	40

**Table 3:** Parameter estimation results for resonant frequency degradation simulation data, comparing joint estimation and separate estimation with preset parameters.

Parameter	Joint Estimation		Average Independent Estimation		Difference Between Separate and Joint Estimation
	Estimated Value	Difference from Preset Parameters	Estimated Value	Difference from Preset Parameters	
$\mu_{f}$	8.70 × 10^−2^	1.51%	8.71 × 10^−2^	1.63%	0.11%
$\sigma_{f}^{2}$	1.27	5.83%	1.00	16.7%	21.3%

**Table 4:** Parameter estimation results from simulated Q-factor degradation data.

Parameter	Estimated Value	Simulation Preset Parameter Values	Difference
$\alpha_{0}$	14.43 × 10^−5^	14.48 × 10^−5^	0.35%
$\alpha_{1}$	20.04 × 10^−5^	20.40 × 10^−5^	1.76%
$\alpha_{D}$	5.91 × 10^−3^	6.00 × 10^−3^	1.50%
$\sigma_{Q}$	8.99 × 10^−8^	1.00 × 10^−7^	10.10%

## Data Availability

The data that support the findings of this study are available from the corresponding author upon reasonable request.

## Abbreviations

The following abbreviations are used in this manuscript:

Bi-LSTM	Bidirectional Long Short-Term Memory
CNN	Convolutional Neural Network
DCFP	Dependent Competing Failure Process
DRSN-TCN	Deep Residual Shrinkage Network–Temporal Convolutional Network
IMU	Inertial Measurement Unit
LSTM	Long Short-Term Memory
MEMS	Micro-Electro-Mechanical System
MLE	Maximum Likelihood Estimation
OLS	Ordinary Least Squares
RUL	Remaining Useful Life
VRG	Vibrating Ring Gyroscope
xLSTM	Extended Long Short-Term Memory

## References

[1] Zeng J., Liu Z., Liu C. (2025). Research on Roll Attitude Estimation Algorithm for Precision Firefighting Extinguishing Projectiles Based on Single MEMS Gyroscope. Sensors.

[2] Huang S., Zhou Y., Ke Z., Li Y., Liu M., Zhang L., Jiang B., Liu F., Su Y. (2025). Collaborative Force Rebalance Strategy for Enhanced Dynamic Range and Resolution in MEMS Gyroscopes. IEEE Trans. Instrum. Meas..

[3] Luo H., Su H., Tang Q., Nisa F.U., He L., Zhang T., Liu X., Liu Z. (2025). Review of Research Advances in Gyroscopes’ Structural Forms and Processing Technologies Viewed from Performance Indices. Sensors.

[4] Zhou P., Chen J., Zhang P., Ge Y., Ren X., Han B., Lin X., Nan L. (2026). A Magnetic Beacon and MEMS-IMUs Array Cable Fused Positioning Method for Subsea Stratum Drilling Robots. Meas. Sci. Technol..

[5] Gołkowski M., Kwaśniewski J., Roskosz M., Mazurek P., Molski S., Grzybowski J. (2024). Use of Attitude and Heading Reference System (AHRS) to Analyze the Impact of Safety Nets on the Accelerations Occurring in the Human Body During a Collision. Sensors.

[6] Carratù M., Gallo V., Dello Iacono S., Sommella P., Ciani L., Patrizi G. (2025). A New Health Index for RUL Estimation of MEMS Sensors Using Dimensionality Reduction and Artificial Neural Networks. IEEE Trans. Instrum. Meas..

[7] Gill W.A., Howard I., Mazhar I., McKee K. (2022). A Review of MEMS Vibrating Gyroscopes and Their Reliability Issues in Harsh Environments. Sensors.

[8] Bu Z., Long B., Liu Z., Wu K., Geng H., Cheng Y. (2025). Multivariate Adaptive Brownian Motion-Particle Filter Framework for Remaining Useful Life Prediction of Nonlinear and State-Noise Coupled Degradation Process. Reliab. Eng. Syst. Saf..

[9] Wu J., Liu Y., Wang H., Ma X., Zhao Y. (2025). A Novel Mechanism-Equivalence-Based Tweedie Exponential Dispersion Process for Adaptive Degradation Modeling and Life Prediction. Sensors.

[10] Wu J., Su S., Li P., Wang L., Yan S., Han S. (2025). A Thermo-Wear Dual-Parameter Wiener Model Approach for Predicting the Remaining Useful Life of Wet Friction Components. Meas. Sci. Technol..

[11] Dong Q., Pei H., Hu C., Zheng J., Du D. (2025). Remaining Useful Life Prediction Method for Stochastic Degrading Devices Considering Predictive Maintenance. Sensors.

[12] Li W., Chen J., Chen S., Li P., Zhang B., Wang M., Yang M., Wang J., Zhou D., Yun J. (2025). A Comprehensive Review of Artificial Intelligence-Based Algorithms for Predicting the Remaining Useful Life of Equipment. Sensors.

[13] Wang P., Li M., Wang C., Li X., Duan L., Di R., Lv Z. (2026). Novel Integrated Health Indicator and DRSN-TCN Based Remaining Useful Life Prediction for Rolling Bearings. Meas. Sci. Technol..

[14] Li F., Dai Z., Jiang L., Song C., Zhong C., Chen Y. (2024). Prediction of the Remaining Useful Life of Bearings Through CNN-Bi-LSTM-Based Domain Adaptation Model. Sensors.

[15] Jiang R., Li Z., Lu H., Mo W., Huang W., Xu M. (2026). RUL Prediction Based on xLSTM–Transformer Neural Network for Rolling Element Bearings Under Different Working Conditions. Sensors.

[16] Chen X., Liu Z. (2022). A Long Short-Term Memory Neural Network Based Wiener Process Model for Remaining Useful Life Prediction. Reliab. Eng. Syst. Saf..

[17] Li J., Broas M., Makkonen J., Mattila T.T., Hokka J., Paulasto-Krockel M. (2014). Shock Impact Reliability and Failure Analysis of a Three-Axis MEMS Gyroscope. J. Microelectromech. Syst..

[18] Xu Y., Liu S., He C., Wu H., Cheng L., Huang Q., Yan G. (2023). Research on Packaging Reliability and Quality Factor Degradation Model for Wafer-Level Vacuum Sealing MEMS Gyroscopes. Micromachines.

[19] Wang J., Cai Q., Wei W., Cui R., Shi Y., Shen C., Cao H. (2024). Failure Mechanism Analysis and Experiment of MEMS VRG Under High-g Shock. IEEE Sens. J..

[20] Liu Y., Zhang S., Hou Z., Fan Z., Wang Y., Peng X., Chen X. (2020). An Investigate on Degradation Models of Resonant Frequency and Mechanical Sensitivity for Butterfly Resonator Gyroscope. J. Microelectromech. Syst..

[21] Cheng J., Qian Z., Li Z. (2021). A Cumulative Fatigue Damage Model of Polysilicon Films for MEMS Resonator under Repeated Loadings. Int. J. Fatigue.

[22] Cheng J., Chen X., Li Z., Lu J., Liu B. (2024). Quantitative Analysis of Performance Degradation in Movable MEMS Devices by a Multiscale Approach. Eng. Fail. Anal..

[23] Cheng J., Li G., Shen H., Dai L. (2023). Failure Analysis from Microcracks to a Dominant Crack in MEMS Thin Films Using Combined Damage and Fracture Mechanics. Eng. Fail. Anal..

[24] Sadurska W.L., Imboden M., Burger J., Dommann A.J. (2025). Advancing Understanding of High-Temperature Micro-Electro-Mechanical System Failures with New Simulation-Assisted Approach. Sensors.

[25] Satheesh S.M., Banerjee A., Bhattacharya E. (2021). Transferability of Weakest Link Model Parameters of Polysilicon from Indentation Fracture to Failure of MEMS Structures. Eng. Fail. Anal..

[26] Chen Z., Yan K., Wang X., Li R., Zhang A., Wang X., Wang Y., Gao P., Li H., Wang C. (2025). MEMS Gyroscopes in Different Operation Modes: A Review. Measurement.

[27] Zhang H., Zhang C., Chen J., Li A. (2022). A Review of Symmetric Silicon MEMS Gyroscope Mode-Matching Technologies. Micromachines.

[28] Bu F., Fan B., Feng R., Zhou M., Wang Y. (2025). Automatic Mode-Matching Method for MEMS Gyroscope Based on Fast Mode Reversal. Micromachines.

[29] Ren J., Zhou T., Zhou Y., Li Y., Su Y. (2024). An Automatic Q-Factor Matching Method for Eliminating 77% of the ZRO of a MEMS Vibratory Gyroscope in Rate Mode. Microsyst. Nanoeng..

[30] Bashir U., Bazaz S.A., Saleem M.M., Shakoor R.I., Tariq M.O., Kumar P. (2024). Design and Analysis of Mode-Matched Decoupled Mass MEMS Gyroscope with Improved Thermal Stability. Meas. Sci. Technol..

[31] Cui J., Yan G., Zhao Q. (2019). Enhanced Temperature Stability of Scale Factor in MEMS Gyroscope Based on Multi Parameters Fusion Compensation Method. Measurement.

[32] Wang J., Wang R., Han X. (2023). Degradation Modeling and Reliability Estimation for Competing Risks Considering System Resistance. Comput. Ind. Eng..

[33] Chang M., Coolen F.P.A., Coolen-Maturi T., Huang X. (2024). A Generalized System Reliability Model Based on Survival Signature and Multiple Competing Failure Processes. J. Comput. Appl. Math..

[34] Tanner D.M., Dugger M.T. (2003). Wear Mechanisms in a Reliability Methodology (Invited). Reliability, Testing, and Characterization of MEMS/MOEMS II.

[35] Zhang S., Zhang J. (2021). Fatigue-Induced Dynamic Pull-in Instability in Electrically Actuated Microbeam Resonators. Int. J. Mech. Sci..

[36] Alter A.L., Flader I.B., Chen Y., Ortiz L.C., Shin D.D., Kenny T.W. (2020). Characterization of Accelerated Fatigue in Thick Epi-Polysilicon Vacuum Encapsulated MEMS Resonators. J. Microelectromech. Syst..

[37] Dong E., Gao T., Cheng Z., Wang R., Bai Y. (2022). Opportunistic Maintenance Strategy for Complex Equipment with a Genetic Algorithm Considering Failure Dependence: A Two-Dimensional Warranty Perspective. Sensors.

[38] Zhang J.-A., Chen Y., Wang Y., Li Y., Kang R. (2026). Reliability Modeling and Evaluation of Complex Electronic Systems with Multi-Group DCFPs. Reliab. Eng. Syst. Saf..

[39] Liu M., Yao X., Zhang J., Chen W., Jing X., Wang K. (2020). Multi-Sensor Data Fusion for Remaining Useful Life Prediction of Machining Tools by IABC-BPNN in Dry Milling Operations. Sensors.

[40] Yin Y., Gao Z., Huang Y., Liu Q. (2025). PLT-GRU: Physics-Informed Lightweight Transformer-GRU Algorithm for Few-Shot Battery State-of-Health Estimation. Meas. Sci. Technol..

[41] Wu J., Zhou Y., Wang X., Chen C., Ma Y., Zhang C. (2026). Research on Remaining Useful Life Prediction of Equipment Based on Digital Twins. Sensors.

[42] Shaveta, Bhan R.K., Chaujar R. (2026). Beyond Planar: A Vertical Sense Mass Approach to Overcome Damping Challenge in MEMS Gyroscope. Micro Nanostruct..

[43] Schiwietz D., Weig E.M., Degenfeld-Schonburg P. (2023). Thermoelastic Damping in MEMS Gyroscopes at High Frequencies. Microsyst. Nanoeng..

[44] Muhlstein C.L., Howe R.T., Ritchie R.O. (2004). Fatigue of Polycrystalline Silicon for Microelectromechanical System Applications: Crack Growth and Stability under Resonant Loading Conditions. Mech. Mater..

[45] Muhlstein C.L., Brown S.B., Ritchie R.O. (2001). High-Cycle Fatigue and Durability of Polycrystalline Silicon Thin Films in Ambient Air. Sens. Actuators Phys..

[46] Muhlstein C.L., Stach E.A., Ritchie R.O. (2002). A Reaction-Layer Mechanism for the Delayed Failure of Micron-Scale Polycrystalline Silicon Structural Films Subjected to High-Cycle Fatigue Loading. Acta Mater..

[47] Connally J.A., Brown S.B. (1992). Slow Crack Growth in Single-Crystal Silicon. Science.

[48] Kahn H., Ballarini R., Bellante J.J., Heuer A.H. (2002). Fatigue Failure in Polysilicon Not Due to Simple Stress Corrosion Cracking. Science.

[49] Li P., Fang Y. (2015). An Analytical Model for Squeeze-Film Damping of Perforated Torsional Microplates Resonators. Sensors.

[50] Wang A., Sahandabadi S., Harrison T., Spicer D., Ahamed M.J. (2022). Modelling of Air Damping Effect on the Performance of Encapsulated MEMS Resonators. Microsyst. Technol..

